# The Immuno‐Cardiovascular Storm of Pre‐Eclampsia: Cytokine‐Mediated Endothelial Injury, Thrombosis, and Maternal Cardiovascular Instability

**DOI:** 10.1002/cph4.70209

**Published:** 2026-06-30

**Authors:** Emmanuel Ifeanyi Obeagu

**Affiliations:** ^1^ Division of Haematology, Department of Biomedical and Laboratory Science Africa University Mutare Zimbabwe; ^2^ Department of Molecular Medicine and Haematology, Faculty of Health Sciences University of the Witwatersrand Johannesburg South Africa

**Keywords:** cytokine storm, endothelial dysfunction, immunothrombosis, maternal cardiovascular instability, pre‐eclampsia

## Abstract

Pre‐eclampsia is a complex multisystem hypertensive disorder of pregnancy and a leading cause of maternal and perinatal morbidity and mortality worldwide. Increasing evidence suggests that the condition extends beyond abnormal placentation and represents an integrated immuno‐cardiovascular disorder characterized by immune dysregulation, endothelial dysfunction, thromboinflammation, and cardiovascular maladaptation. This narrative review examines the interconnected mechanisms linking cytokine‐mediated inflammation, endothelial injury, coagulation abnormalities, and maternal cardiovascular instability in pre‐eclampsia. A structured literature search of major biomedical databases was conducted to identify relevant experimental, clinical, and translational studies addressing inflammatory cytokines, angiogenic imbalance, endothelial dysfunction, thrombosis, biomarkers, and therapeutic strategies in pre‐eclampsia. The review highlights the central roles of pro‐inflammatory mediators, including tumor necrosis factor‐α, interleukin‐6, interleukin‐17, and interferon‐γ, in promoting oxidative stress, vascular inflammation, and endothelial dysfunction. Particular attention is given to the development of an immunothrombotic environment, defined as the pathological interaction between immune activation and coagulation pathways that promotes thrombosis and vascular injury. The review also critically evaluates the contribution of antiangiogenic factors, platelet activation, impaired fibrinolysis, and cardiovascular remodeling to maternal disease progression. In addition, current and emerging therapeutic approaches targeting inflammatory, endothelial, angiogenic, and thrombotic pathways are discussed, together with their translational potential and clinical limitations. By integrating immunological, vascular, hematological, and cardiovascular perspectives, this review provides a comprehensive framework for understanding the pathophysiology of pre‐eclampsia and identifies important knowledge gaps for future investigation.

## Graphical abstract

Placental ischemia in pre‐eclampsia initiates cytokine‐driven endothelial dysfunction, angiogenic imbalance, oxidative stress, and thromboinflammation. Elevated TNF‐α, IL‐6, IL‐17, sFlt‐1, and complement activation promote vasoconstriction, thrombosis, and cardiovascular instability. Progressive vascular injury culminates in maternal hypertension, organ dysfunction, microvascular ischemia, adverse fetal outcomes, and long‐term cardiovascular risk.

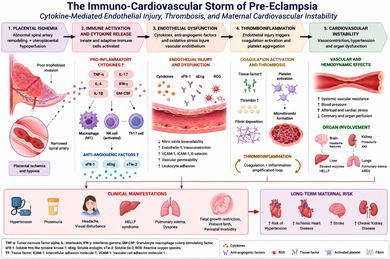

## 1 Introduction

Pre‐eclampsia is a multifactorial, pregnancy‐specific hypertensive disorder characterized by new‐onset hypertension after 20 weeks of gestation accompanied by proteinuria and/or evidence of maternal organ dysfunction, uteroplacental dysfunction, or both. Despite substantial advances in obstetric care, pre‐eclampsia remains one of the leading causes of maternal and perinatal morbidity and mortality worldwide, affecting approximately 2%–8% of pregnancies and accounting for a significant proportion of maternal deaths, preterm births, fetal growth restriction, and neonatal complications. The burden of the disease is particularly pronounced in low‐ and middle‐income countries, where limitations in antenatal surveillance, diagnostic capabilities, and access to specialized obstetric care contribute to adverse outcomes. Beyond its immediate obstetric consequences, pre‐eclampsia has emerged as a major predictor of long‐term cardiovascular, cerebrovascular, and metabolic disease in affected women, highlighting its significance as both a pregnancy complication and a lifelong cardiovascular risk factor (Uzan et al. 2011; Phipps et al. 2019; Bisson et al. 2023). Historically, pre‐eclampsia was viewed primarily as a placental disorder resulting from defective trophoblast invasion and inadequate remodeling of maternal spiral arteries. According to the widely accepted two‐stage model, abnormal placentation leads to reduced uteroplacental perfusion, placental ischemia, oxidative stress, and the release of pathogenic factors into the maternal circulation. These placental‐derived mediators subsequently induce systemic endothelial dysfunction, hypertension, and multisystem organ injury. Although this model remains central to our understanding of disease pathogenesis, increasing evidence suggests that it does not fully explain the complexity and heterogeneity of pre‐eclampsia. Clinical variability among patients, differences between early‐onset and late‐onset disease, and inconsistencies in biomarker expression indicate the involvement of additional biological mechanisms extending beyond placental pathology alone (Bakrania et al. 2020; Hod et al. 2015; Yang, Tian, et al. 2022).

Recent investigations have increasingly recognized pre‐eclampsia as a disorder arising from complex interactions among the immune, vascular, coagulation, and cardiovascular systems. Pregnancy normally requires the establishment of a highly regulated immunological balance that permits maternal tolerance of the semi‐allogeneic fetus while preserving effective host defense mechanisms. Successful gestation is characterized by coordinated interactions among trophoblasts, uterine natural killer cells, dendritic cells, macrophages, regulatory T cells, and numerous cytokines that collectively support placental development and maternal‐fetal immune tolerance. In pre‐eclampsia, however, this delicate balance is disrupted, resulting in exaggerated maternal immune activation, loss of immunological tolerance, increased inflammatory cytokine production, and widespread vascular injury (Liu et al. 2024; Possomato‐Vieira and Khalil 2016). A growing body of evidence demonstrates that women with pre‐eclampsia exhibit heightened activation of both innate and adaptive immune pathways. Elevated circulating concentrations of pro‐inflammatory cytokines, including tumor necrosis factor‐alpha (TNF‐α), interleukin (IL)‐6, IL‐1β, IL‐17, and interferon‐gamma (IFN‐γ), have been consistently reported in affected pregnancies. These inflammatory mediators contribute to endothelial activation, oxidative stress, vascular dysfunction, and abnormal coagulation. Simultaneously, reductions in regulatory T‐cell activity and alterations in immune cell populations promote a pro‐inflammatory environment that perpetuates disease progression. Importantly, although inflammatory activation is a well‐established feature of pre‐eclampsia, the extent to which it resembles a classical “cytokine storm” remains controversial. Unlike acute cytokine storm syndromes observed in severe infections or immune‐mediated conditions, pre‐eclampsia appears to involve a sustained and dysregulated inflammatory response of varying intensity rather than a universally defined cytokine storm. Consequently, careful interpretation of inflammatory terminology is required when describing the immunopathology of the disease (Sánchez‐Aranguren et al. 2014; Costantini et al. 2024; Chu 2011).

Endothelial dysfunction represents the principal downstream consequence of these inflammatory and placental disturbances and serves as a central pathogenic mechanism linking placental injury to maternal clinical manifestations. The vascular endothelium regulates vascular tone, coagulation, permeability, leukocyte trafficking, and angiogenesis. In pre‐eclampsia, endothelial cells undergo profound functional alterations characterized by reduced nitric oxide bioavailability, increased endothelin‐1 production, enhanced oxidative stress, expression of adhesion molecules, and increased vascular permeability. These changes promote vasoconstriction, hypertension, tissue hypoperfusion, and multiorgan dysfunction. Endothelial injury is further exacerbated by circulating antiangiogenic factors, particularly soluble fms‐like tyrosine kinase‐1 (sFlt‐1) and soluble endoglin (sEng), which antagonize vascular endothelial growth factor (VEGF) and placental growth factor (PlGF), thereby impairing vascular homeostasis and endothelial repair mechanisms (Kutllovci Hasani et al. 2025; Gongora and Wenger 2015). In addition to endothelial dysfunction, increasing attention has focused on the role of thromboinflammation and immunothrombosis in the pathogenesis of pre‐eclampsia. Immunothrombosis refers to the bidirectional interaction between immune and coagulation pathways whereby inflammatory activation promotes thrombosis and thrombotic processes amplify inflammation. Placental ischemia and systemic inflammatory mediators stimulate platelet activation, tissue factor expression, thrombin generation, and fibrin deposition while simultaneously impairing fibrinolytic pathways. These alterations create a prothrombotic state characterized by microvascular thrombosis, endothelial injury, and organ ischemia. The concept of immunothrombosis provides an important mechanistic framework for understanding how inflammatory and coagulation pathways converge to drive disease progression and contribute to complications such as HELLP syndrome, disseminated intravascular coagulation, placental infarction, and adverse fetal outcomes (Lamarca 2010).

Cardiovascular involvement represents another critical yet often underappreciated component of pre‐eclampsia pathophysiology. Normal pregnancy is associated with extensive cardiovascular adaptation, including increased plasma volume, enhanced cardiac output, reduced systemic vascular resistance, and structural remodeling of the maternal heart. In contrast, increased vascular resistance, impaired arterial compliance, left ventricular remodeling, diastolic dysfunction, myocardial strain, and heightened susceptibility to heart failure and arrhythmias characterize pre‐eclampsia. These cardiovascular abnormalities not only contribute to acute maternal complications during pregnancy but also persist beyond delivery in many women, increasing the risk of future hypertension, ischemic heart disease, stroke, heart failure, and cardiovascular mortality. Consequently, pre‐eclampsia is increasingly recognized as a cardiovascular disease risk marker that reveals underlying susceptibility to vascular dysfunction later in life (Gathiram and Moodley 2016). Although numerous reviews have examined individual aspects of pre‐eclampsia, including placental pathology, immune dysfunction, angiogenic imbalance, endothelial injury, or cardiovascular complications, relatively few have comprehensively integrated these interconnected mechanisms into a unified pathophysiological framework. Existing literature often evaluates inflammatory, thrombotic, endothelial, and cardiovascular processes independently, despite substantial evidence that these systems interact dynamically throughout disease progression. Furthermore, recent advances in immunology, vascular biology, and cardio‐obstetrics have generated new insights into the molecular pathways linking placental ischemia to maternal systemic disease, creating a need for an updated synthesis of current evidence.

The present narrative review seeks to address this knowledge gap by critically examining the interconnected pathways that drive the immunological, endothelial, thrombotic, and cardiovascular manifestations of pre‐eclampsia. Specifically, this review explores how cytokine‐mediated immune dysregulation contributes to endothelial injury, how inflammatory and coagulation pathways interact to create an immunothrombotic environment, and how these processes culminate in maternal cardiovascular instability. In addition, the review evaluates biomarker development, disease heterogeneity, current and emerging therapeutic strategies, and the translational implications of targeting the immuno‐cardiovascular axis. By integrating evidence from immunology, vascular biology, hematology, obstetrics, and cardiovascular medicine, this review proposes the concept of an “immuno‐cardiovascular storm” as a conceptual framework describing the complex and self‐amplifying interactions among inflammatory, endothelial, thrombotic, and cardiovascular pathways in pre‐eclampsia. Rather than implying a classical cytokine storm syndrome, this framework serves to illustrate the multidimensional and interconnected nature of disease pathogenesis and its profound impact on maternal health.

## 2 Methods

### 2.1 Literature Search Strategy and Study Selection

This narrative review was conducted to synthesize current evidence regarding the immunological, endothelial, thrombotic, and cardiovascular mechanisms underlying pre‐eclampsia, with particular emphasis on cytokine‐mediated endothelial injury, immunothrombosis, and maternal cardiovascular instability. Although the review was not designed as a systematic review or meta‐analysis, a structured and comprehensive literature search was undertaken to ensure the inclusion of relevant and high‐quality evidence. A literature search was performed across multiple electronic databases, including PubMed/MEDLINE, Scopus, Web of Science, Embase, and Google Scholar, covering publications from January 2000 to March 2026. Additional relevant articles were identified through manual screening of reference lists from eligible studies, review articles, clinical guidelines, and landmark publications. The search strategy combined Medical Subject Headings (MeSH) terms and free‐text keywords related to pre‐eclampsia and its immuno‐cardiovascular manifestations. Search terms included combinations of: “pre‐eclampsia,” “preeclampsia,” “immune dysregulation,” “inflammation,” “cytokines,” “tumor necrosis factor‐alpha,” “interleukin‐6,” “interleukin‐17,” “interferon‐gamma,” “endothelial dysfunction,” “angiogenic imbalance,” “soluble fms‐like tyrosine kinase‐1,” “soluble endoglin,” “immunothrombosis,” “thromboinflammation,” “coagulation,” “platelet activation,” “maternal cardiovascular adaptation,” “cardiac remodeling,” “cardiovascular disease,” and “pregnancy‐associated hypertension.”

The review included original research articles, observational studies, cohort studies, case–control studies, translational investigations, experimental studies, systematic reviews, meta‐analyses, consensus statements, and evidence‐based clinical guidelines that addressed one or more aspects of the immunological, endothelial, coagulation, or cardiovascular pathways involved in pre‐eclampsia. Preference was given to studies directly investigating pre‐eclampsia populations, placental biology, maternal vascular dysfunction, inflammatory signaling pathways, biomarker development, and therapeutic interventions. Highly cited seminal studies and recent publications with significant translational or clinical relevance were prioritized to ensure comprehensive coverage of the evolving literature. Articles were excluded if they were conference abstracts without full‐text availability, duplicate publications, editorials lacking substantive scientific analysis, non‐peer‐reviewed reports, studies unrelated to pre‐eclampsia pathophysiology, or publications with insufficient methodological information. Only articles published in English were considered for inclusion. Studies focusing exclusively on general inflammatory or cardiovascular mechanisms without direct relevance to pre‐eclampsia were evaluated carefully and included only when they provided essential mechanistic insights applicable to disease pathogenesis.

Following database searches and manual screening procedures, an initial pool of publications was identified and assessed for relevance based on titles, abstracts, and full‐text evaluation. The final review incorporated 74 articles, comprising original research studies, clinical investigations, systematic reviews, meta‐analyses, and international guideline documents. These studies were selected based on their scientific quality, methodological rigor, relevance to the review objectives, and contribution to understanding the complex interactions among immune activation, endothelial dysfunction, thrombosis, and cardiovascular instability in pre‐eclampsia. The included literature was narratively synthesized and organized into major thematic areas, including abnormal placentation and immune dysregulation, cytokine‐mediated endothelial injury, angiogenic imbalance, immunothrombosis and coagulation abnormalities, cardiovascular maladaptation, biomarker development, therapeutic interventions, disease heterogeneity, and long‐term cardiovascular consequences. Particular emphasis was placed on critically evaluating mechanistic pathways, identifying areas of scientific consensus and controversy, highlighting translational implications, and discussing emerging directions for future research. This approach allowed for an integrated and multidisciplinary examination of the immuno‐cardiovascular mechanisms that contribute to the pathogenesis and clinical manifestations of pre‐eclampsia.

### 2.2 Pathophysiological Foundations of the Immuno‐Cardiovascular Storm

Pre‐eclampsia is increasingly understood not merely as a hypertensive disorder of pregnancy but as a complex immuno‐cardiovascular syndrome arising from intertwined placental, immune, and vascular dysfunctions. The pathophysiological foundations of this syndrome are rooted in abnormal placentation during early gestation, which initiates a cascade of events that amplify maternal systemic inflammation, endothelial injury, and cardiovascular instability (Singh 2009). Normal placentation involves the invasion of maternal spiral arteries by extravillous cytotrophoblasts, transforming these vessels into low‐resistance conduits capable of supporting the increasing demands of the growing fetus. In pre‐eclampsia, this process is incomplete, resulting in shallow trophoblastic invasion and inadequate remodeling of the spiral arteries. The consequence is placental hypoperfusion and intermittent ischemia, which generate oxidative stress and release of soluble factors into the maternal circulation. Among these, antiangiogenic molecules such as soluble fms‐like tyrosine kinase‐1 (sFlt‐1) and soluble endoglin antagonize vascular endothelial growth factor (VEGF) and transforming growth factor‐beta (TGF‐β) signaling. This imbalance not only impairs angiogenesis but also destabilizes endothelial homeostasis, setting the stage for systemic vascular dysfunction (Lyall et al. 2001; Pollheimer et al. 2018).

Placental ischemia also triggers a pronounced maternal immune response, characterized by the release of pro‐inflammatory cytokines including tumor necrosis factor‐alpha (TNF‐α), interleukin‐6 (IL‐6), interleukin‐1β (IL‐1β), and interferon‐gamma (IFN‐γ). These cytokines orchestrate a systemic inflammatory environment that resembles a controlled cytokine storm, driving widespread endothelial activation and injury. Activated endothelial cells express adhesion molecules, recruit leukocytes, and increase vascular permeability, creating a milieu conducive to vascular inflammation and thrombosis (LaMarca et al. 2016; Carpentier et al. 2011). The inflammatory activation extends to coagulation pathways. Cytokines stimulate tissue factor expression on endothelial cells and monocytes, while simultaneously promoting platelet activation and impairing fibrinolysis. This phenomenon, referred to as immunothrombosis, represents a convergence of immune and coagulation mechanisms, where the body's attempt to contain inflammation paradoxically causes microvascular thrombi and vascular obstruction. These thrombotic events exacerbate endothelial injury and contribute to organ dysfunction observed in severe pre‐eclampsia, including the kidney, liver, and brain (Wilhelm et al. 2023; Costantini et al. 2024). The combined effects of placental ischemia, immune activation, endothelial dysfunction, and thrombotic microvascular injury culminate in maternal cardiovascular instability. Systemic vasoconstriction, increased arterial stiffness, and altered cardiac output characterize the hemodynamic manifestations of the disease. Persistent inflammatory and thrombotic insults can lead to myocardial stress, cardiac remodeling, and long‐term predisposition to cardiovascular disease, highlighting the far‐reaching consequences of the immuno‐cardiovascular storm beyond pregnancy (Table 1) (Gilbert et al. 2008; Schwartz and Stanhewicz 2024).

**TABLE 1:** Pathophysiological foundations of the immuno‐cardiovascular storm in pre‐eclampsia.

Pathophysiological component	Principal mechanisms	Key molecular mediators	Maternal consequences	Clinical/translational significance	Key references
Abnormal placentation	Inadequate trophoblast invasion and incomplete spiral artery remodeling leading to placental hypoperfusion and ischemia–reperfusion injury	HIF‐1α, VEGF dysregulation, placental oxidative stress mediators	Placental ischemia, release of pathogenic placental factors	Initiating event in many cases of early‐onset pre‐eclampsia; target for early prediction strategies	Redman and Sargent (2005), Staff (2019)
Placental oxidative stress	Excessive reactive oxygen species (ROS) production and mitochondrial dysfunction resulting from intermittent placental hypoxia	ROS, NADPH oxidase, lipid peroxidation products	Endothelial activation, cellular injury, systemic inflammation	Potential target for antioxidant and mitochondrial‐directed therapies	Burton et al. (2019), Rana et al. (2019)
Immune dysregulation	Loss of maternal‐fetal immune tolerance with exaggerated innate and adaptive immune activation	Reduced Tregs, activated NK cells, Th1 and Th17 responses	Chronic inflammatory state, impaired placental development	Supports development of immune‐based biomarkers and therapeutic targets	Laresgoiti‐Servitje (2013), Cornelius (2018)
Pro‐inflammatory cytokine activation	Increased production of inflammatory mediators amplifying endothelial dysfunction and vascular injury	TNF‐α, IL‐6, IL‐1β, IL‐17, IFN‐γ	Hypertension, vascular inflammation, oxidative stress	May contribute to disease severity and biomarker development	Harmon et al. (2016), Mol et al. (2016)
Angiogenic imbalance	Excess antiangiogenic factor production with suppression of endothelial repair pathways	sFlt‐1, soluble endoglin (sEng), ↓PlGF, ↓VEGF bioavailability	Endothelial dysfunction, hypertension, proteinuria	Established diagnostic and prognostic biomarker pathway	Maynard et al. (2003), Zeisler et al. (2016)
Endothelial dysfunction	Loss of vascular homeostasis with impaired vasodilation and increased vascular permeability	Endothelin‐1, reduced nitric oxide (NO), VCAM‐1, ICAM‐1	Vasoconstriction, organ hypoperfusion, hypertension	Central mediator linking placental pathology to maternal disease	Roberts and Hubel (2009), Phipps et al. (2019)
Immunothrombosis	Bidirectional interaction between inflammation and coagulation pathways promoting thrombus formation	Tissue factor, thrombin, NETs, complement proteins	Microvascular thrombosis, placental infarction, organ dysfunction	Emerging therapeutic target linking inflammation and coagulation	Raghupathy (2013), Witkowski et al. (2026)
Platelet activation	Enhanced platelet aggregation and release of pro‐inflammatory and procoagulant mediators	Thromboxane A_2_, P‐selectin, soluble CD40 ligand	Hypercoagulability, endothelial injury	Biological rationale for low‐dose aspirin prophylaxis	Roberge et al. (2018), Sharma et al. (2024)
Impaired fibrinolysis	Reduced breakdown of fibrin due to elevated fibrinolytic inhibitors	PAI‐1, PAI‐2, fibrin degradation products	Persistent thrombosis and vascular occlusion	Contributes to disease progression and thrombotic complications	Mayrink et al. (2018)
Complement system activation	Excessive activation of innate immune pathways leading to endothelial damage	C3a, C5a, membrane attack complex (C5b‐9)	Vascular injury, inflammation, thrombosis	Emerging biomarker and therapeutic pathway	Lokki et al. (2018)
Extracellular vesicle signaling	Placental‐derived extracellular vesicles transfer inflammatory and antiangiogenic signals to maternal tissues	Syncytiotrophoblast extracellular vesicles, microRNAs, DAMPs	Systemic inflammation and endothelial activation	Promising area for biomarker discovery and mechanistic studies	Tannetta et al. (2017), Turé et al. (2024)
Cardiovascular maladaptation	Failure of normal maternal cardiovascular adaptation with increased vascular resistance and cardiac stress	Endothelin‐1, inflammatory cytokines, antiangiogenic factors	Left ventricular remodeling, diastolic dysfunction, heart failure risk	Explains both acute maternal instability and long‐term cardiovascular risk	Melchiorre et al. (2014), Carola et al. (2024)
Long‐term cardiovascular programming	Persistent endothelial and vascular dysfunction following affected pregnancies	Chronic inflammation, vascular remodeling pathways	Increased risk of hypertension, stroke, ischemic heart disease, and heart failure	Supports lifelong cardiovascular surveillance after pre‐eclampsia	Wu et al. (2017), Morales‐Suarez‐Varela and Guillen‐Grima (2025)

Abbreviations: DAMPs, damage‐associated molecular patterns; HIF‐1α, hypoxia‐inducible factor‐1 alpha; ICAM‐1, intercellular adhesion molecule‐1; IFN‐γ, interferon‐gamma; IL, interleukin; NETs, neutrophil extracellular traps; NK, natural killer cell; NO, nitric oxide; PAI, plasminogen activator inhibitor; PlGF, placental growth factor; sEng, soluble endoglin; sFlt‐1, soluble fms‐like tyrosine kinase‐1; TNF‐α, tumor necrosis factor‐alpha; Treg, regulatory T cell; VCAM‐1, vascular cell adhesion molecule‐1; VEGF, vascular endothelial growth factor.

### 2.3 Cytokine Storm and Immune Dysregulation in Pre‐Eclampsia

Pre‐eclampsia is increasingly recognized as a disorder of immune dysregulation, in which an exaggerated maternal inflammatory response plays a central role in disease pathogenesis. At the heart of this process is a cytokine storm–like environment, characterized by elevated circulating levels of pro‐inflammatory mediators that disrupt vascular homeostasis and contribute to systemic maternal morbidity (Michalczyk et al. 2020; Collier et al. 2021). Under normal pregnancy conditions, a tightly regulated balance between pro‐inflammatory and anti‐inflammatory immune responses ensures fetal tolerance while maintaining maternal defense against infection. In pre‐eclampsia, this equilibrium is lost. Placental ischemia and oxidative stress generate danger signals that activate maternal innate and adaptive immune cells, including monocytes, neutrophils, and T lymphocytes. These activated cells release high levels of cytokines such as tumor necrosis factor‐α (TNF‐α), interleukin‐6 (IL‐6), interleukin‐1β (IL‐1β), and interferon‐γ (IFN‐γ), amplifying systemic inflammation. The magnitude and persistence of this inflammatory response resemble a controlled cytokine storm, which has profound implications for endothelial function, coagulation, and cardiovascular stability (Zhang 2008; Cook‐Mills et al. 2011).

The cytokine surge induces endothelial activation, marked by increased expression of adhesion molecules such as ICAM‐1 and VCAM‐1, which recruit leukocytes to the vascular wall and promote vascular inflammation. It also disrupts endothelial nitric oxide signaling, increasing vascular tone and contributing to hypertension. Simultaneously, cytokine‐mediated activation of platelets and monocytes enhances tissue factor expression, accelerating the coagulation cascade and establishing a prothrombotic state—a process now described as immunothrombosis, wherein immune and coagulation pathways intersect to contain inflammatory insults but inadvertently cause vascular damage (Pfister 2022; Zhou et al. 2022). Neutrophil extracellular traps (NETs) and extracellular vesicles further amplify the inflammatory and thrombotic milieu. NETs provide a scaffold for platelet aggregation and fibrin deposition, while extracellular vesicles carry inflammatory and procoagulant molecules that propagate endothelial injury at distant sites. This interplay between cytokine signaling, immune cell activation, and endothelial perturbation establishes a self‐perpetuating loop, intensifying maternal vascular dysfunction and increasing the risk of severe complications such as eclampsia, HELLP syndrome, and multi‐organ failure (Table 2) (Powe et al. 2011; Lamarca 2012).

**TABLE 2:** Cytokine‐mediated immune dysregulation (“cytokine storm–like” response) in pre‐eclampsia.

Immune axis/cytokine	Source/cellular origin	Key mechanistic actions in pre‐eclampsia	Downstream vascular and placental effects	Clinical correlates	Evidence strength/notes	Key references
TNF‐α (tumor necrosis factor‐α)	Activated macrophages, trophoblasts, NK cells	Induces endothelial activation, promotes oxidative stress, reduces NO bioavailability	Vasoconstriction, endothelial injury, increased vascular permeability	Severity of hypertension, proteinuria	Strong association; consistent elevation in PE cohorts	Harmon et al. (2016), Lan et al. (2022)
IL‐6 (interleukin‐6)	Monocytes/macrophages, adipose tissue, placenta	Drives acute‐phase response, enhances endothelial activation, promotes coagulation signaling	Elevated CRP, endothelial dysfunction, platelet activation	Severe disease phenotypes, metabolic‐linked PE	Moderate–strong evidence; correlated with severity	Jannesari and Kazemi (2017), Cornelius (2018)
IL‐1β (interleukin‐1 beta)	Inflammasome‐activated macrophages, trophoblasts	Promotes endothelial adhesion molecule expression, amplifies inflammation	Leukocyte adhesion, vascular inflammation, oxidative injury	Associated with inflammatory phenotypes	Moderate evidence; mechanistic support from models	Hoshino et al. (2021)
IL‐17 (interleukin‐17)	Th17 lymphocytes	Enhances neutrophil recruitment, stimulates ROS production, amplifies cytokine cascades	Endothelial injury, vascular inflammation, hypertension	Severe PE, immune‐skewed phenotypes	Emerging but growing evidence base	Plug et al. (2025), McGeachy et al. (2019)
IFN‐γ (interferon‐gamma)	Th1 cells, NK cells	Inhibits trophoblast invasion, promotes macrophage activation, increases antigen presentation	Impaired placentation, endothelial dysfunction	Early‐onset and severe PE	Moderate evidence; strong mechanistic plausibility	Laresgoiti‐Servitje (2013)
IL‐10 (anti‐inflammatory cytokine)	Tregs, trophoblasts, monocytes	Suppresses inflammatory cytokine production; maintains immune tolerance	Loss of immunoregulation when reduced	Reduced levels associated with disease progression	Strong inverse association in PE	Su et al. (2016)
TGF‐β (transforming growth factor‐β)	Tregs, platelets, placenta	Regulates immune tolerance, endothelial repair, and vascular stability	Endothelial dysfunction when dysregulated	Linked to abnormal immune tolerance	Mixed evidence; context‐dependent effects	Horvat Mercnik et al. (2024)
Th1/Th2 imbalance	Adaptive immune system	Shift toward Th1 dominance increases pro‐inflammatory signaling	Placental injury, systemic inflammation	Early‐onset and severe PE phenotypes	Well‐established immunological pattern	Saito and Sakai (2003)
Th17/Treg imbalance	CD4+ T cell subsets	Increased Th17 and reduced Treg function leads to immune activation	Endothelial injury, chronic inflammation	Severe PE, recurrent PE risk	Strong emerging immunological framework	Cornelius (2018)
Chemokines (e.g., MCP‐1, IL‐8)	Endothelial cells, macrophages	Recruit monocytes and neutrophils to vascular endothelium	Leukocyte infiltration, vascular inflammation	Associated with endothelial dysfunction severity	Moderate evidence	Roy et al. (2014)
Inflammasome activation (NLRP3)	Macrophages, placental trophoblasts	Activates IL‐1β and IL‐18 signaling pathways	Amplified systemic inflammation	Emerging mechanism in severe PE	Early mechanistic evidence	Geng et al. (2023)
Complement–cytokine crosstalk	Innate immune system	Amplifies cytokine release via C3a/C5a signaling	Endothelial injury, thrombosis	Severe PE, HELLP syndrome	Increasing experimental and clinical evidence	Lokki et al. (2024)
Systemic “cytokine storm–like” state (conceptual integration)	Multi‐cellular immune activation	Combined elevation of pro‐inflammatory mediators with endothelial activation	Immunothrombosis, vascular dysfunction, multiorgan injury	Severe PE phenotypes, critical illness	Conceptual framework; not classical cytokine storm	Schneller‐Pavelescu et al. (2026)

Abbreviations: CRP, C‐reactive protein; IFN‐γ, interferon‐gamma; IL, interleukin; MCP‐1, monocyte chemoattractant protein‐1; NLRP3, NOD‐like receptor pyrin domain‐containing protein 3; NO, nitric oxide; PE, pre‐eclampsia; ROS, reactive oxygen species; Th, T helper cell; TNF‐α, tumor necrosis factor‐alpha; Treg, regulatory T cell.

### 2.4 Cytokine‐Mediated Endothelial Injury

A central feature of pre‐eclampsia is endothelial dysfunction, which emerges as both a consequence and a driver of the systemic inflammatory environment. Endothelial cells line the vascular system and maintain vascular tone, barrier integrity, and hemostatic balance. In pre‐eclampsia, excessive cytokine release from the ischemic placenta and activated maternal immune cells initiates a cascade of events that compromise these critical endothelial functions (Schuerwegh et al. 2003; Kany et al. 2019). Pro‐inflammatory cytokines—including tumor necrosis factor‐alpha (TNF‐α), interleukin‐6 (IL‐6), interleukin‐1β (IL‐1β), and interferon‐gamma (IFN‐γ)—play pivotal roles in this process. TNF‐α increases endothelial permeability and promotes leukocyte adhesion, while IL‐6 acts as a key mediator linking inflammation to coagulation and vascular remodeling. IL‐1β facilitates local vascular inflammation by inducing expression of adhesion molecules such as ICAM‐1 and VCAM‐1, which recruit circulating leukocytes to the endothelial surface. The combined effect is a pro‐inflammatory, pro‐adhesive endothelial phenotype that disrupts vascular homeostasis (Muth et al. 2023; Ogunlola et al. 2019; Aggarwal et al. 2012).

In parallel, antiangiogenic factors released from the placenta, including soluble fms‐like tyrosine kinase‐1 (sFlt‐1) and soluble endoglin, impair VEGF and TGF‐β signaling, further destabilizing endothelial integrity. This antiangiogenic environment reduces nitric oxide bioavailability, diminishes vasodilation, and increases vascular reactivity, setting the stage for systemic hypertension. Oxidative stress generated both by ischemic placental tissue and activated leukocytes compounds endothelial injury by damaging cellular membranes, mitochondria, and the glycocalyx—a protective barrier that regulates vascular permeability (Palomo et al. 2023; Theofilis et al. 2021; Harmon et al. 2016). The result of these combined insults is endothelial activation and dysfunction, which is clinically manifested as hypertension, proteinuria, and capillary leak. Moreover, endothelial injury triggers the release of microparticles and procoagulant molecules that amplify the immunothrombotic cascade, linking vascular inflammation to platelet activation and microvascular thrombosis. This self‐perpetuating cycle of cytokine‐mediated endothelial injury and thrombotic activation contributes not only to the acute manifestations of pre‐eclampsia but also to long‐term cardiovascular remodeling, increasing maternal susceptibility to future vascular disease (Table 3) (Deer et al. 2023; Oladosu‐Olayiwola et al. 2018).

**TABLE 3:** Cytokine‐mediated endothelial injury in pre‐eclampsia.

Pro‐inflammatory mediator/trigger	Primary source	Endothelial cellular pathways affected	Functional endothelial consequences	Vascular/organ‐level outcomes	Clinical correlates in pre‐eclampsia	Evidence strength	Key references
TNF‐α	Macrophages, placenta, NK cells	NF‐κB activation, ROS generation, apoptosis signaling	↓ NO bioavailability, ↑ permeability, endothelial apoptosis	Vasoconstriction, microvascular injury	Hypertension severity, proteinuria	Strong	Lan et al. (2022), Harmon et al. (2016)
IL‐6	Monocytes, adipose tissue, placenta	JAK/STAT3 signaling, acute‐phase activation	↑ CRP production, endothelial activation, procoagulant shift	Systemic inflammation, vascular stiffness	Severe disease, metabolic‐associated PE	Strong–moderate	Jannesari and Kazemi (2017)
IL‐1β	Inflammasome‐activated macrophages	NLRP3 inflammasome pathway, NF‐κB amplification	↑ adhesion molecules (VCAM‐1, ICAM‐1), leukocyte adhesion	Endothelial inflammation, capillary dysfunction	Severe inflammatory phenotypes	Moderate	Geng et al. (2023)
IL‐17	Th17 cells	ROS amplification, neutrophil recruitment pathways	Endothelial oxidative stress, barrier disruption	Hypertension, vascular inflammation	Early‐onset and severe PE	Emerging–strong	Cornelius (2018)
IFN‐γ	Th1 cells, NK cells	JAK/STAT1 signaling, immune activation	Impaired trophoblast‐endothelial interaction, endothelial activation	Placental ischemia, vascular dysfunction	Early‐onset PE	Moderate	Hoshino et al. (2021)
sFlt‐1 (antiangiogenic trigger)	Placenta (syncytiotrophoblast)	VEGF/PlGF sequestration → endothelial VEGF deprivation	↓ endothelial repair, ↓ NO production	Widespread endothelial dysfunction	Diagnostic marker; correlates with severity	Strong	Maynard et al. (2003), Zeisler et al. (2016)
Soluble endoglin (sEng)	Placenta	TGF‐β pathway inhibition	Impaired endothelial regeneration, ↑ vascular permeability	Severe endothelial injury	Severe PE, HELLP syndrome	Strong	Venkatesha et al. (2006)
Reactive oxygen species (ROS)	Mitochondrial dysfunction, NADPH oxidase	Oxidative stress pathways, lipid peroxidation	Endothelial apoptosis, NO scavenging	Vasospasm, organ ischemia	Severe PE progression	Strong	Burton et al. (2019)
Complement activation (C5a, C3a)	Innate immune system	Membrane attack complex formation, leukocyte activation	Endothelial lysis, inflammation amplification	Microvascular thrombosis	HELLP syndrome, severe PE	Emerging–moderate	Lokki et al. (2024)
NETs (neutrophil extracellular traps)	Activated neutrophils	Histone‐mediated endothelial toxicity	Endothelial damage, thrombosis promotion	Placental infarction, vascular occlusion	Severe PE, immunothrombotic phenotype	Emerging	Gupta and Kaplan (2016)
Platelet‐derived mediators (TXA_2_, sCD40L)	Activated platelets	Platelet–endothelium interaction pathways	Vasoconstriction, endothelial activation	Hypercoagulability, vascular injury	Disease severity, thrombosis risk	Strong	Roberge et al. (2018)
Antiangiogenic–inflammatory synergy (integrated pathway)	Placenta + immune cells	Combined VEGF inhibition + cytokine signaling	Profound endothelial dysfunction, barrier failure	Multiorgan endothelial injury	Severe/early‐onset PE	Strong conceptual	Schneller‐Pavelescu et al. (2026)

Abbreviations: ICAM‐1, intercellular adhesion molecule‐1; IFN‐γ, interferon‐gamma; IL, interleukin; JAK/STAT, Janus kinase/signal transducer and activator of transcription pathway; NETs, neutrophil extracellular traps; NF‐κB, nuclear factor kappa‐light‐chain‐enhancer of activated B cells; NO, nitric oxide; PE, pre‐eclampsia; PlGF, placental growth factor; ROS, reactive oxygen species; sCD40L, soluble CD40 ligand; sEng, soluble endoglin; sFlt‐1, Soluble fms‐like tyrosine kinase‐1; TNF‐α, tumor necrosis factor‐alpha; TXA_2_, thromboxane A2; VCAM‐1, vascular cell adhesion molecule‐1; VEGF, vascular endothelial growth factor.

### 2.5 Immunothrombosis and Coagulation Activation

A critical aspect of the immuno‐cardiovascular storm in pre‐eclampsia is the interplay between inflammation and coagulation, a phenomenon now conceptualized as immunothrombosis. In this process, cytokine‐driven immune activation and endothelial injury converge to create a hypercoagulable environment that contributes to microvascular dysfunction and maternal organ injury (Martini et al. 2025; Mora‐Palazuelos et al. 2022). The cytokine surge characteristic of pre‐eclampsia—primarily involving TNF‐α, IL‐6, and IL‐1β—induces the expression of tissue factor on endothelial cells and circulating monocytes. Tissue factor serves as a critical initiator of the extrinsic coagulation pathway, leading to thrombin generation, fibrin formation, and the activation of platelets. Concurrently, activated platelets release pro‐inflammatory mediators that further amplify endothelial activation and leukocyte recruitment, reinforcing the cycle of inflammation and coagulation (Stefańska et al. 2021; Martini et al. 2025; Chen et al. 2021). In addition to cellular activation, pre‐eclampsia is associated with impaired anticoagulant pathways, including reduced protein C and antithrombin activity, and suppression of fibrinolytic mechanisms due to elevated plasminogen activator inhibitor‐1 (PAI‐1). The cumulative effect is a prothrombotic state that favors microvascular thrombosis in critical maternal organs, including the kidneys, liver, brain, and placenta. Microthrombi reduce perfusion, exacerbate endothelial injury, and contribute to the development of severe complications such as HELLP syndrome, eclampsia, and multi‐organ dysfunction (Caillon et al. 2022; Mutua and Gershwin 2021).

**FIGURE 1:**
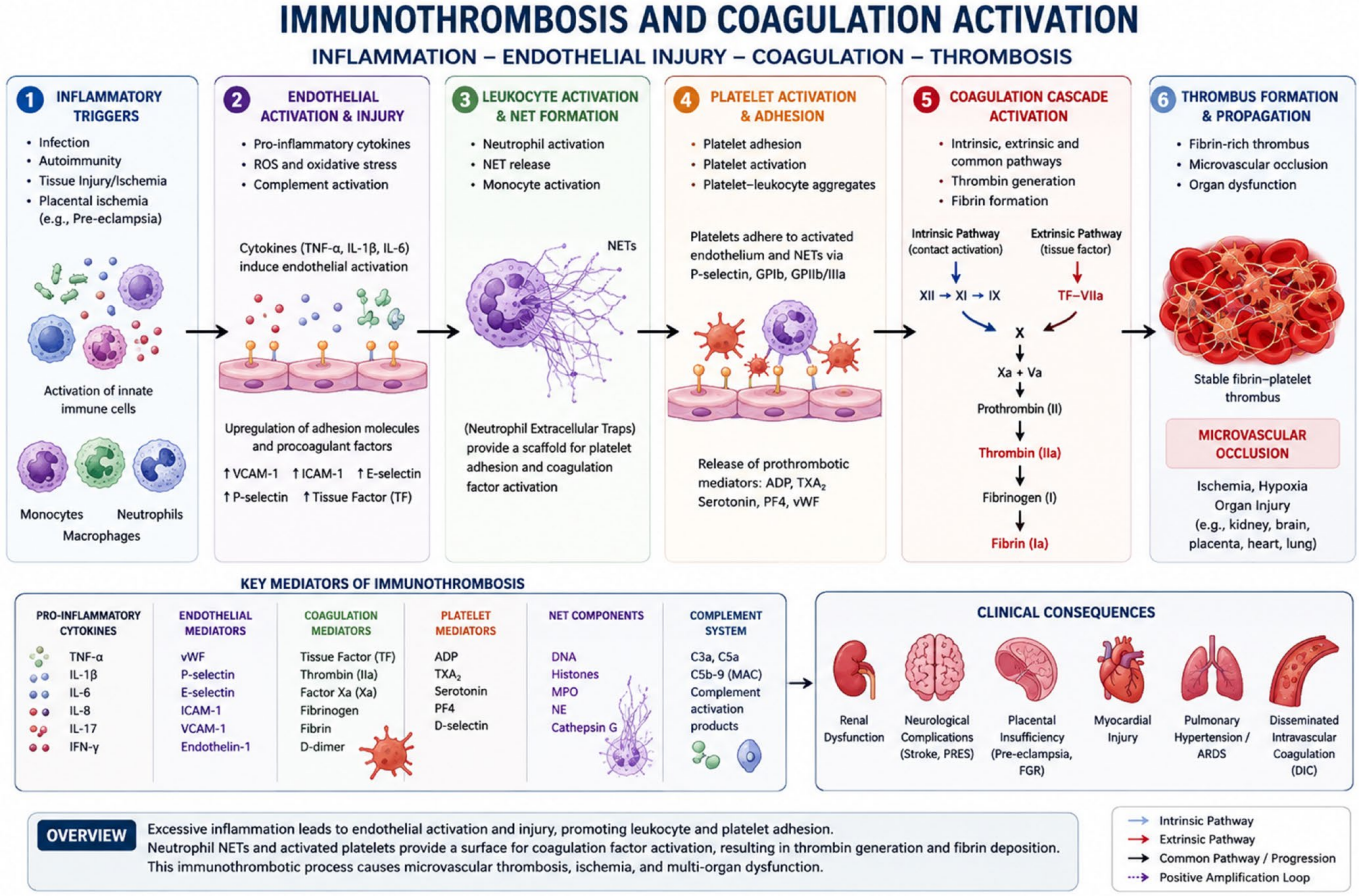
Immunothrombosis and coagulation activation.

Emerging evidence also highlights the role of neutrophil extracellular traps (NETs) and extracellular vesicles in the propagation of immunothrombosis. NETs provide a structural scaffold that enhances platelet aggregation and fibrin deposition, while extracellular vesicles carry tissue factor and inflammatory cytokines, amplifying both coagulation and endothelial damage at distant vascular sites (Gathiram and Moodley 2016; Palmiero et al. 2025; Wang and He 2024). Thus, immunothrombosis in pre‐eclampsia represents a mechanistic bridge between immune dysregulation and cardiovascular instability. By linking inflammation to coagulation, it not only contributes to the acute clinical manifestations of the disease but also sets the stage for long‐term vascular remodeling and maternal cardiovascular risk. Recognizing the central role of immunothrombosis provides a conceptual basis for developing therapies that target both inflammation and coagulation, offering potential avenues to mitigate maternal morbidity and improve outcomes in pre‐eclamptic pregnancies (Figure 1) (Sun et al. 2020; Saheera and Krishnamurthy 2020).

### 2.6 Cardiovascular Instability and Maternal Hemodynamic Consequences

The culmination of cytokine‐mediated endothelial injury and immunothrombosis in pre‐eclampsia is maternal cardiovascular instability, which represents both an acute clinical challenge and a predictor of long‐term vascular risk. The systemic inflammatory and thrombotic environment disrupts normal vascular homeostasis, leading to hemodynamic alterations that compromise organ perfusion and increase cardiovascular strain (Saheera and Krishnamurthy 2020; Mărgulescu et al. 2012). Endothelial dysfunction, driven by pro‐inflammatory cytokines and antiangiogenic placental factors, reduces nitric oxide bioavailability, impairs vasodilation, and increases vascular reactivity. Elevated levels of circulating cytokines and oxidative stress further enhance arterial stiffness and systemic vascular resistance, creating a high‐pressure environment that challenges maternal cardiac function. Concurrent immunothrombotic processes, including microvascular thrombosis and platelet aggregation, exacerbate vascular obstruction, impair tissue perfusion, and contribute to organ ischemia (Chávez and Cavalli 2016; Shaw et al. 2024).

**FIGURE 2:**
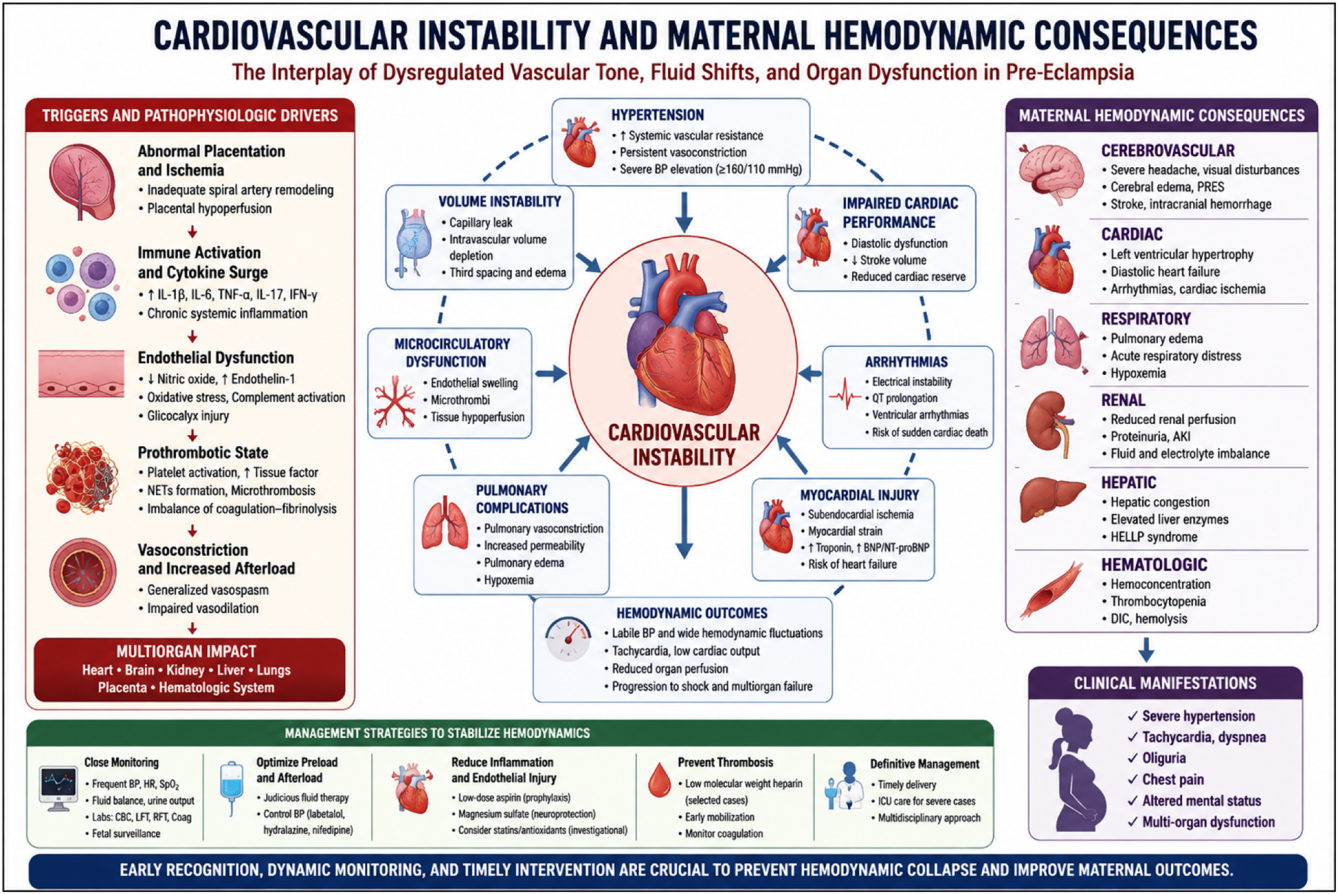
Cardiovascular instability and maternal hemodynamic consequences.

Cardiac adaptations to these vascular changes include increased afterload, diastolic dysfunction, and myocardial stress, which can manifest clinically as hypertension, pulmonary edema, or acute heart failure in severe cases. Additionally, chronic exposure to this inflammatory and thrombotic milieu may promote cardiac remodeling, including left ventricular hypertrophy and fibrosis, which can persist postpartum and predispose women to long‐term cardiovascular disease (Kornacki et al. 2021; Ullmo et al. 2026). Hemodynamic instability in pre‐eclampsia is further compounded by alterations in intravascular volume, increased capillary permeability, and endothelial glycocalyx disruption. These changes lead to fluid shifts, edema, and decreased effective circulating volume despite hypertension, contributing to a paradoxical state of vascular overload and organ hypoperfusion. The combined effects of vascular, inflammatory, and thrombotic perturbations create a self‐reinforcing cycle of cardiovascular compromise, which is central to the maternal morbidity and mortality associated with severe pre‐eclampsia (Figure 2) (Tomkiewicz and Darmochwał‐Kolarz 2024; Amer et al. 2025).

### 2.7 Phenotypic Heterogeneity of Pre‐Eclampsia

Pre‐eclampsia is increasingly recognized as a heterogeneous syndrome rather than a single disease entity. Although traditionally defined by the common clinical features of hypertension and maternal organ dysfunction arising after 20 weeks of gestation, substantial variation exists in disease onset, severity, placental involvement, maternal cardiovascular adaptation, immunological profiles, biomarker expression, and clinical outcomes. This heterogeneity has important implications for disease pathogenesis, diagnosis, risk stratification, therapeutic intervention, and long‐term maternal health. Contemporary evidence suggests that multiple biological pathways may converge to produce the clinical phenotype of pre‐eclampsia, resulting in distinct subtypes with varying underlying mechanisms and prognostic significance (Amer et al. 2025). One of the most clinically relevant classifications distinguishes early‐onset pre‐eclampsia (EOPE) from late‐onset pre‐eclampsia (LOPE). Early‐onset disease, typically defined as pre‐eclampsia requiring delivery before 34 weeks of gestation, accounts for a smaller proportion of cases but is associated with more severe maternal and fetal complications. EOPE is strongly linked to defective placentation, inadequate spiral artery remodeling, placental hypoperfusion, oxidative stress, and pronounced angiogenic imbalance. Women with EOPE generally exhibit significantly elevated circulating levels of soluble fms‐like tyrosine kinase‐1 (sFlt‐1), reduced placental growth factor (PlGF), heightened inflammatory activation, and more extensive endothelial dysfunction compared with normotensive pregnancies. Placental lesions, fetal growth restriction, and adverse perinatal outcomes are also more prevalent in this subgroup. Consequently, EOPE is often considered the placental‐driven form of the disease, where placental pathology serves as the primary initiator of systemic maternal manifestations (Udomsinprasert et al. 2021).

In contrast, late‐onset pre‐eclampsia, occurring at or beyond 34 weeks of gestation, is generally more common and exhibits a different pathophysiological profile. While placental abnormalities may still be present, maternal constitutional factors such as obesity, metabolic syndrome, insulin resistance, chronic hypertension, advanced maternal age, and pre‐existing cardiovascular dysfunction appear to play a more prominent role. Compared with EOPE, LOPE is frequently characterized by less severe angiogenic imbalance and relatively preserved placental morphology. However, maternal cardiovascular maladaptation, systemic inflammation, endothelial dysfunction, and metabolic disturbances remain important contributors to disease development. Emerging evidence suggests that LOPE may represent a maternal cardiovascular susceptibility syndrome in which pregnancy acts as a physiological stress test that unmasks pre‐existing vascular dysfunction (Lyall et al. 1994). Differences in immunological profiles further support the concept of distinct pre‐eclampsia phenotypes. Several studies have demonstrated that women with EOPE exhibit higher concentrations of pro‐inflammatory cytokines, greater activation of innate immune pathways, and more pronounced alterations in regulatory T‐cell populations compared with those with LOPE. Elevated levels of TNF‐α, IL‐6, IL‐17, and other inflammatory mediators have been associated with severe placental insufficiency and adverse maternal outcomes. However, findings across studies remain inconsistent, reflecting differences in study populations, disease definitions, timing of sample collection, and laboratory methodologies. These observations underscore the need for standardized approaches to immunophenotyping in future investigations (Budak et al. 1998).

The distinction between mild and severe pre‐eclampsia provides another important dimension of disease heterogeneity. Severe disease is associated with higher blood pressure levels, greater degrees of endothelial dysfunction, more extensive coagulation abnormalities, and increased risks of complications such as HELLP syndrome, eclampsia, acute kidney injury, pulmonary edema, and maternal death. Women with severe pre‐eclampsia often exhibit markedly elevated concentrations of inflammatory cytokines, antiangiogenic factors, and markers of oxidative stress compared with those with milder disease. Nevertheless, progression from mild to severe disease is not universal, suggesting that additional genetic, immunological, environmental, and cardiovascular factors influence disease trajectory (Yang, Feng, et al. 2022). Increasing attention has also focused on the existence of angiogenic and non‐angiogenic phenotypes of pre‐eclampsia. The angiogenic phenotype is characterized by significant elevations in sFlt‐1, reductions in PlGF, severe placental dysfunction, and poorer maternal‐fetal outcomes. In contrast, some women meet clinical diagnostic criteria for pre‐eclampsia despite relatively normal angiogenic profiles. These observations suggest that alternative pathogenic pathways, including maternal cardiovascular dysfunction, metabolic disturbances, systemic inflammation, or genetic susceptibility, may contribute to disease development independently of profound angiogenic imbalance. Recognition of these phenotypes may help explain differences in clinical presentation and response to therapeutic interventions (Sayyadi et al. 2023).

Another emerging concept is the distinction between placental pre‐eclampsia and maternal pre‐eclampsia. Placental pre‐eclampsia is predominantly driven by abnormal placental development, severe uteroplacental insufficiency, and fetal growth restriction, whereas maternal pre‐eclampsia appears to arise primarily from pre‐existing maternal cardiovascular, metabolic, or inflammatory vulnerabilities. Although these categories are not mutually exclusive, they provide a useful framework for understanding the diverse biological pathways contributing to disease expression. Importantly, many patients likely exhibit overlapping features of both forms, reflecting the multifactorial nature of the syndrome (Munguía et al. 2022). Genetic and epigenetic factors further contribute to phenotypic diversity. Variations in genes regulating angiogenesis, immune function, oxidative stress, coagulation, and vascular homeostasis have been implicated in susceptibility to pre‐eclampsia. Epigenetic modifications affecting placental and maternal gene expression may also influence disease severity and clinical phenotype. However, the precise contribution of genetic factors remains incompletely understood and continues to be an active area of investigation (Sharma et al. 2024).

From a cardiovascular perspective, substantial heterogeneity exists in maternal hemodynamic adaptation. Some women exhibit predominantly high‐resistance, low‐cardiac‐output states characterized by marked vasoconstriction and endothelial dysfunction, whereas others demonstrate high‐cardiac‐output phenotypes associated with obesity, metabolic syndrome, and volume overload. These differences may have important implications for individualized treatment strategies and long‐term cardiovascular risk assessment. Recent studies have suggested that distinct cardiovascular phenotypes may influence responsiveness to antihypertensive therapy and predict future cardiovascular disease risk following pregnancy (Rider et al. 2016). The recognition of phenotypic heterogeneity has important clinical and research implications. It challenges the traditional view of pre‐eclampsia as a uniform disorder and supports the development of precision medicine approaches tailored to specific biological subtypes. Improved characterization of inflammatory, angiogenic, thrombotic, metabolic, and cardiovascular phenotypes may facilitate earlier diagnosis, enhance risk prediction, identify novel therapeutic targets, and improve maternal‐fetal outcomes. Furthermore, understanding disease heterogeneity may help explain the variable performance of biomarkers and therapeutic interventions observed across clinical studies (Table 4) (Ren et al. 2023).

**TABLE 4:** Major phenotypic variants of pre‐eclampsia and their characteristics.

Phenotype	Key pathophysiological features	Biomarker profile	Maternal outcomes	Fetal outcomes
Early‐onset pre‐eclampsia (< 34 weeks)	Defective placentation, severe placental ischemia, angiogenic imbalance	↑ sFlt‐1, ↓ PlGF, ↑ inflammatory cytokines	Severe hypertension, HELLP syndrome, organ dysfunction	Fetal growth restriction, prematurity, perinatal mortality
Late‐onset pre‐eclampsia (≥ 34 weeks)	Maternal cardiovascular and metabolic dysfunction predominates	Mild‐to‐moderate angiogenic changes	Hypertension, cardiovascular stress	Generally better fetal outcomes
Mild pre‐eclampsia	Limited endothelial dysfunction	Moderate biomarker abnormalities	Lower complication risk	Usually favorable outcomes
Severe pre‐eclampsia	Marked endothelial injury, inflammation, coagulation activation	High cytokine levels, high sFlt‐1/PlGF ratio	HELLP syndrome, eclampsia, pulmonary edema	Increased fetal morbidity
Angiogenic phenotype	Significant placental dysfunction	Markedly elevated sFlt‐1/PlGF ratio	Higher disease severity	Higher risk of fetal growth restriction
Non‐angiogenic phenotype	Maternal cardiovascular/metabolic factors predominate	Near‐normal angiogenic markers	Variable severity	Variable fetal effects
Placental pre‐eclampsia	Placental insufficiency‐driven disease	Strong angiogenic imbalance	Severe maternal manifestations	Significant fetal compromise
Maternal pre‐eclampsia	Underlying cardiovascular/metabolic susceptibility	Variable biomarker changes	Increased long‐term cardiovascular risk	Less pronounced placental dysfunction

### 2.8 Emerging Biomarkers of the Immuno‐Cardiovascular Storm

The identification of reliable biomarkers is increasingly recognized as a critical step in understanding, diagnosing, and managing the immuno‐cardiovascular storm in pre‐eclampsia. These biomarkers provide insight into the interconnected processes of inflammation, endothelial dysfunction, and immunothrombosis, and may facilitate early risk stratification, monitoring of disease progression, and evaluation of therapeutic interventions (Amer et al. 2025; Udomsinprasert et al. 2021). Cytokines and inflammatory mediators remain central to the biomarker landscape. Elevated circulating levels of tumor necrosis factor‐alpha (TNF‐α), interleukin‐6 (IL‐6), interleukin‐1β (IL‐1β), and interferon‐gamma (IFN‐γ) reflect systemic immune activation and correlate with disease severity. Measurement of these cytokines can provide a window into the magnitude of the maternal inflammatory response, offering both diagnostic and prognostic value (Lyall et al. 1994; Budak et al. 1998; Yang, Feng, et al. 2022).

Markers of endothelial activation and injury also serve as critical indicators of the vascular consequences of pre‐eclampsia. Soluble adhesion molecules, such as ICAM‐1 and VCAM‐1, and circulating endothelial microparticles reflect endothelial perturbation and the loss of vascular integrity. Antiangiogenic factors, including soluble fms‐like tyrosine kinase‐1 (sFlt‐1) and soluble endoglin, indicate disruption of VEGF and TGF‐β signaling, correlating with both hypertension and systemic vascular instability. Additionally, measures of endothelial glycocalyx degradation, such as syndecan‐1, are emerging as potential markers of vascular barrier compromise (Sayyadi et al. 2023; Munguía et al. 2022).

**FIGURE 3:**
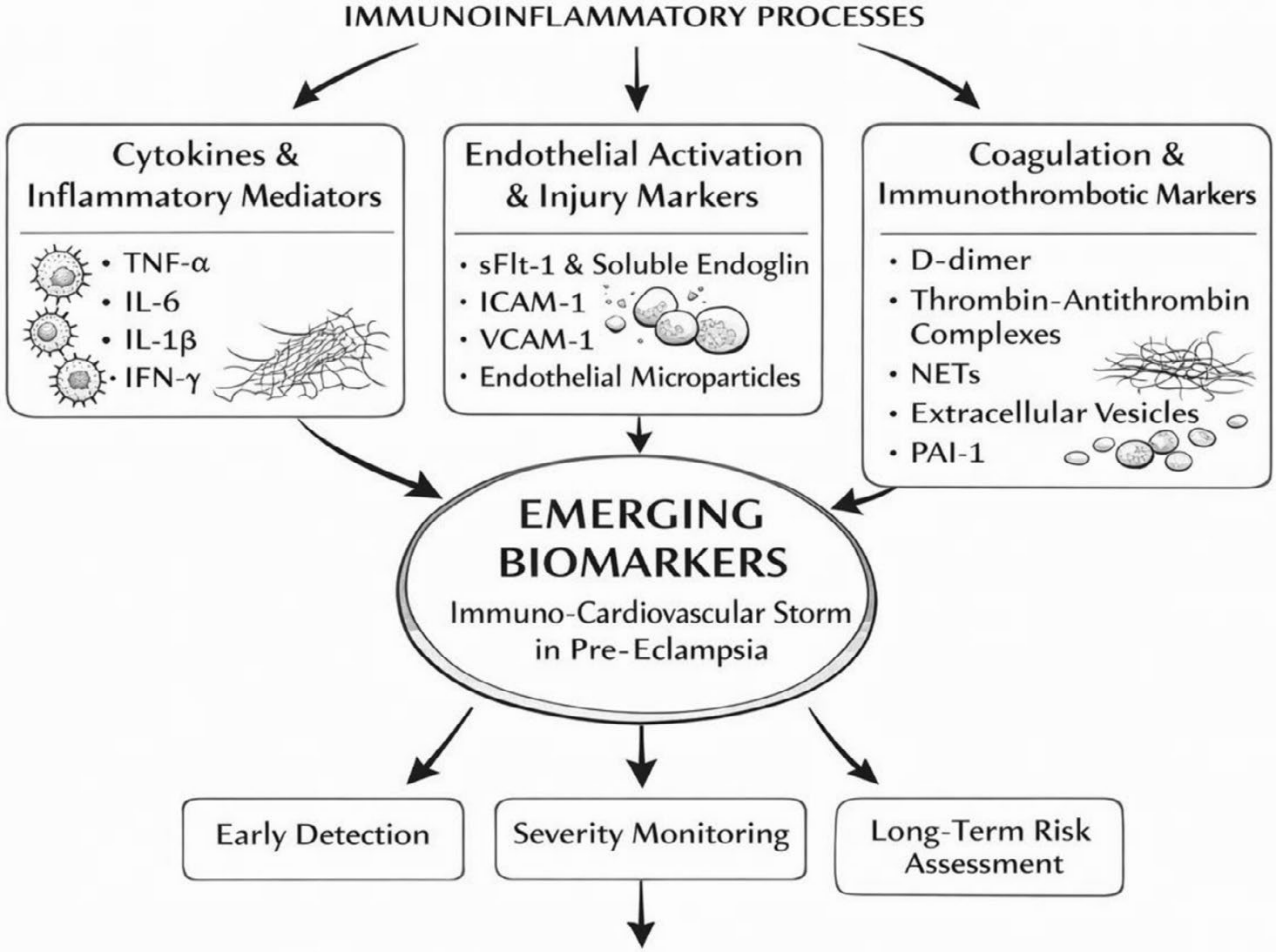
Emerging biomarkers of the immuno‐cardiovascular storm.

Coagulation and immunothrombotic markers further expand the biomarker profile. Elevated D‐dimer, thrombin‐antithrombin complexes, and plasminogen activator inhibitor‐1 (PAI‐1) reflect activation of the coagulation cascade, platelet involvement, and impaired fibrinolysis. Moreover, neutrophil extracellular traps (NETs) and extracellular vesicles carrying tissue factor or inflammatory mediators have been identified as circulating indicators of immunothrombosis, linking immune activation directly to prothrombotic processes (Sharma et al. 2024; Rider et al. 2016). The integration of multimodal biomarkers—encompassing cytokines, endothelial injury markers, coagulation indices, and novel extracellular vesicle signatures—offers a more comprehensive assessment of the immuno‐cardiovascular storm. When combined with clinical parameters such as blood pressure, proteinuria, and cardiac function measures, these biomarkers may enable earlier detection of severe disease, inform individualized management strategies, and predict long‐term cardiovascular risk in affected women (Figure 3) (Ren et al. 2023).

### 2.9 Therapeutic Perspectives

Management of pre‐eclampsia has traditionally focused on *symptomatic control*—primarily blood pressure management, seizure prophylaxis, and timely delivery of the fetus and placenta. However, the emerging understanding of pre‐eclampsia as an *immuno‐cardiovascular storm* highlights new avenues for *targeted therapeutic interventions* aimed at modulating the inflammatory, endothelial, and thrombotic pathways that drive maternal morbidity (Akhigbe 2014).

#### 2.9.1 Anti‐Inflammatory Strategies

Given the central role of cytokine‐mediated immune activation, therapies that *modulate the maternal inflammatory response* have gained attention. Experimental approaches include cytokine‐targeted biologics and small‐molecule inhibitors aimed at reducing TNF‐α, IL‐6, and IL‐1β activity. While clinical translation remains limited, these interventions hold promise for mitigating endothelial injury and dampening the systemic inflammatory cascade without compromising fetal immune tolerance (Kojovic et al. 2020).

#### 2.9.2 Anticoagulation and Antiplatelet Therapy

The recognition of *immunothrombosis* in pre‐eclampsia supports the judicious use of antithrombotic strategies. Low‐dose *aspirin* is already established for high‐risk pregnancies to reduce the incidence of pre‐eclampsia, likely through both antiplatelet and anti‐inflammatory mechanisms. In severe disease, anticoagulants such as *low‐molecular‐weight heparin* are being investigated for their dual ability to inhibit thrombosis and modulate endothelial inflammation. Targeted antiplatelet therapy may further reduce microvascular obstruction and improve maternal organ perfusion (Palo et al. 2025).

#### 2.9.3 Angiogenic Modulation

Placental‐derived *antiangiogenic factors* such as sFlt‐1 play a central role in endothelial dysfunction. Emerging therapies aim to restore angiogenic balance, including *sFlt‐1 neutralization, recombinant placental growth factor supplementation, or apheresis‐based sFlt‐1 reduction*. These strategies seek to preserve endothelial function, reduce vascular resistance, and alleviate maternal hypertension (Chaiworapongsa et al. 2024).

#### 2.9.4 Antioxidant and Endothelial Protective Approaches

Oxidative stress amplifies cytokine signaling and endothelial injury in pre‐eclampsia. Therapeutic strategies targeting oxidative damage—such as *antioxidant supplementation* and agents that stabilize the *endothelial glycocalyx*—may protect vascular integrity and reduce permeability. While clinical evidence remains preliminary, such approaches provide a mechanistic rationale for complementing existing therapies (Palo et al. 2025).

#### 2.9.5 Precision and Multimodal Therapies

Given the heterogeneity of pre‐eclampsia, *precision medicine approaches* integrating maternal biomarkers, cytokine profiles, and vascular function assessments may allow tailored interventions. Combining anti‐inflammatory, antithrombotic, and angiogenic‐modulating therapies could provide *multimodal protection against the immuno‐cardiovascular storm*, potentially improving maternal and fetal outcomes while minimizing risks associated with generalized immunosuppression (Chaiworapongsa et al. 2024).

### 2.10 Maternal–Fetal Safety Considerations in Immunomodulatory and Anticoagulant Therapies for Pre‐Eclampsia

The development of immunomodulatory and anticoagulant strategies for pre‐eclampsia is grounded in the recognition that the disorder involves intersecting pathways of inflammation, endothelial dysfunction, and thromboinflammation. However, translating these mechanistic insights into clinical interventions requires careful consideration of maternal–fetal safety, given the dual physiological unit of pregnancy and the vulnerability of both maternal and fetal compartments to pharmacologic perturbation. At present, most therapies targeting immune or coagulation pathways in pre‐eclampsia remain either prophylactic, adjunctive, or experimental, and their use must be interpreted within a stringent risk–benefit framework. Low‐dose aspirin remains the most widely accepted antithrombotic intervention in pre‐eclampsia prevention. Its primary mechanism involves irreversible inhibition of platelet cyclooxygenase‐1, leading to reduced thromboxane A_2_ production and a shift toward a vasodilatory and antithrombotic state. When initiated early in pregnancy in high‐risk women, low‐dose aspirin has demonstrated modest but consistent reductions in the incidence of pre‐eclampsia, particularly early‐onset disease. Its safety profile is generally favorable, with minimal evidence of significant teratogenicity or major fetal bleeding complications at prophylactic doses. Nevertheless, uncertainties remain regarding optimal dosing, timing of initiation, and patient selection, especially in populations with heterogeneous baseline risk (Ren et al. 2023).

In contrast, more potent anticoagulants such as low‐molecular‐weight heparin (LMWH) are reserved for specific high‐risk indications, including thrombophilias, antiphospholipid syndrome, or recurrent placental‐mediated complications. LMWH does not cross the placenta and is therefore considered relatively safe for the fetus. However, maternal risks include bleeding complications, heparin‐induced thrombocytopenia (rare), osteoporosis with prolonged use, and the need for parenteral administration, which may affect adherence. Importantly, while LMWH has shown benefit in selected high‐risk populations, its routine use for prevention or treatment of pre‐eclampsia without underlying thrombophilia remains unsupported by robust randomized clinical evidence. The potential role of immunomodulatory therapies in pre‐eclampsia, including agents targeting TNF‐α, IL‐6, IL‐17, or complement pathways, is largely confined to experimental and early translational research. Although these pathways are strongly implicated in disease pathophysiology, clinical application in pregnancy remains limited due to substantial safety uncertainties. Biologic agents used in other inflammatory diseases have varying degrees of placental transfer depending on their molecular structure, particularly immunoglobulin G subclasses, raising concerns about fetal exposure during critical periods of immune and organ development. Additionally, suppression of maternal immune responses may theoretically increase susceptibility to infections, impair placental defense mechanisms, and alter fetal immune programming (Akhigbe 2014).

Antiangiogenic‐targeted therapies and endothelial stabilizing strategies are similarly constrained by safety considerations. Interventions aimed at modulating sFlt‐1 or restoring VEGF/PlGF balance hold theoretical promise but raise significant concerns regarding interference with essential angiogenic processes required for placental development, fetal growth, and maternal vascular adaptation. Given the central role of angiogenesis in normal pregnancy, even partial disruption of these pathways could have unintended consequences, including fetal growth restriction or placental insufficiency. Another important safety consideration involves timing of intervention. Many pathogenic processes in pre‐eclampsia are established early in placentation, often before clinical detection. Consequently, late pregnancy interventions may have limited efficacy in reversing established endothelial injury or placental dysfunction, while early interventions carry higher theoretical risks to embryonic and fetal development. This temporal disconnect complicates therapeutic design and emphasizes the importance of preventive rather than purely therapeutic strategies (Kojovic et al. 2020). The maternal cardiovascular system also introduces additional complexity in safety evaluation. Women with pre‐eclampsia frequently exhibit altered hemodynamics, endothelial dysfunction, and increased thrombotic risk. While antithrombotic therapies may theoretically mitigate these processes, excessive anticoagulation or immune suppression may exacerbate hemorrhagic risk or impair adaptive inflammatory responses necessary for pregnancy maintenance. Therefore, any intervention targeting immunothrombotic pathways must balance suppression of pathological inflammation with preservation of physiological immune tolerance and vascular adaptation (Palo et al. 2025).

Fetal safety considerations are equally critical. The placenta functions as the primary interface between maternal therapies and fetal exposure. Drugs that cross the placenta or indirectly alter placental function may affect fetal oxygenation, nutrient transfer, and developmental signaling pathways. Long‐term follow‐up studies evaluating neurodevelopmental, metabolic, and cardiovascular outcomes in exposed offspring remain limited, representing a major gap in current evidence (Chaiworapongsa et al. 2024). A further challenge lies in the heterogeneity of pre‐eclampsia phenotypes. Therapeutic responses and safety profiles may differ substantially between early‐onset and late‐onset disease, as well as between angiogenic and non‐angiogenic subtypes. A “one‐size‐fits‐all” therapeutic approach is therefore unlikely to be appropriate. Instead, precision‐based strategies that account for maternal cardiovascular status, immunological profile, placental function, and biomarker patterns may be required to optimize both efficacy and safety.

### 2.11 Conflicting Evidence, Unresolved Questions, and Limitations of Current Mechanistic Studies

Despite substantial advances in understanding the pathophysiology of pre‐eclampsia, many proposed mechanisms remain incompletely understood, and several aspects of the disease continue to be the subject of ongoing scientific debate. Although numerous studies have identified associations among immune dysregulation, endothelial dysfunction, angiogenic imbalance, thrombosis, and cardiovascular maladaptation, establishing definitive causal relationships remains challenging. Consequently, caution is warranted when interpreting mechanistic findings, particularly because much of the available evidence is derived from observational studies, animal models, in vitro experiments, or relatively small clinical cohorts. One of the most debated issues concerns the role and magnitude of inflammatory activation in pre‐eclampsia. Elevated circulating levels of pro‐inflammatory cytokines such as TNF‐α, IL‐6, IL‐17, and IFN‐γ have been consistently reported in many studies; however, the degree of cytokine elevation varies considerably across investigations. Some reports demonstrate marked increases in inflammatory mediators, whereas others identify only modest differences compared with normotensive pregnancies. These discrepancies may reflect variations in patient populations, disease severity, gestational age at sampling, laboratory methodologies, ethnic backgrounds, and clinical definitions of pre‐eclampsia. Furthermore, unlike classical cytokine storm syndromes observed in severe infections, cytokine concentrations in pre‐eclampsia are generally lower and more variable, raising questions about whether the term “cytokine storm” accurately describes the inflammatory state associated with the disorder. Current evidence may be more consistent with a state of chronic or exaggerated immune activation rather than a true cytokine storm syndrome (Sharma et al. 2024).

The precise role of immune dysregulation in disease initiation also remains uncertain. Although alterations in regulatory T cells, T‐helper cell subsets, natural killer cells, macrophages, and inflammatory cytokines have been documented, it is unclear whether these immune abnormalities represent primary drivers of disease or secondary responses to placental ischemia and tissue injury. Several investigators propose that immune dysfunction precedes placental abnormalities and contributes directly to defective trophoblast invasion, whereas others argue that immune activation occurs predominantly because of placental stress and endothelial injury. Resolving this question remains a major challenge because most human studies evaluate immune parameters after clinical manifestations have already developed (Rider et al. 2016). Similarly, significant controversy exists regarding the relative importance of angiogenic imbalance in the pathogenesis of pre‐eclampsia. Elevated concentrations of sFlt‐1 and reduced levels of PlGF are among the most reproducible findings in the field and have demonstrated considerable utility as diagnostic and prognostic biomarkers. However, not all women with pre‐eclampsia exhibit marked angiogenic abnormalities, and some patients with substantial alterations in angiogenic markers do not develop clinically significant disease. Furthermore, angiogenic profiles differ substantially between early‐onset and late‐onset phenotypes, suggesting that angiogenic imbalance may play a more dominant role in some disease subtypes than others. These observations indicate that sFlt‐1 and PlGF are important contributors to disease pathogenesis but may not fully explain the broad clinical spectrum of pre‐eclampsia (Ren et al. 2023).

Endothelial dysfunction is widely regarded as a central pathogenic feature of pre‐eclampsia; nevertheless, questions remain regarding the specific mechanisms responsible for endothelial injury and the temporal sequence of vascular abnormalities. While antiangiogenic factors, inflammatory cytokines, oxidative stress, extracellular vesicles, and autoantibodies have all been implicated, their relative contributions are difficult to distinguish. Moreover, endothelial dysfunction itself is a heterogeneous phenomenon involving alterations in vascular tone, permeability, coagulation, inflammatory signaling, and angiogenesis. Most available studies assess circulating biomarkers of endothelial activation rather than direct measures of endothelial function, limiting the ability to establish mechanistic causality (Akhigbe 2014). The emerging concept of immunothrombosis has provided valuable insights into the interactions between inflammation and coagulation in pre‐eclampsia. Nevertheless, evidence supporting specific immunothrombotic pathways remains incomplete. While platelet activation, tissue factor expression, fibrin deposition, neutrophil extracellular traps (NETs), and complement activation have been implicated, many studies remain descriptive rather than mechanistic. The extent to which these pathways independently contribute to disease progression, as opposed to serving as markers of underlying vascular injury, remains uncertain. Furthermore, findings regarding complement activation and NET formation have not been entirely consistent across different patient populations and disease phenotypes (Kojovic et al. 2020).

Maternal cardiovascular dysfunction represents another area characterized by both growing interest and unresolved questions. Increasing evidence indicates that women who develop pre‐eclampsia exhibit altered cardiovascular adaptation before clinical diagnosis and remain at elevated cardiovascular risk long after pregnancy. However, whether cardiovascular abnormalities represent a cause, consequence, or combination of both remains unclear. Some investigators propose that pre‐existing subclinical cardiovascular dysfunction predisposes women to pre‐eclampsia, whereas others suggest that placental disease initiates vascular changes that subsequently lead to maternal cardiovascular instability. The likelihood that both pathways contribute to disease development highlights the complexity of the maternal cardiovascular response and underscores the need for longitudinal studies beginning before conception and extending into the postpartum period (Palo et al. 2025). Disease heterogeneity presents an additional challenge in interpreting mechanistic data. Early‐onset and late‐onset pre‐eclampsia differ substantially in their placental pathology, angiogenic profiles, inflammatory responses, cardiovascular characteristics, and clinical outcomes. Similarly, mild and severe disease variants may arise through partially distinct biological pathways. Failure to account for these phenotypic differences contributes to inconsistencies among studies and complicates attempts to identify universal pathogenic mechanisms. Future investigations should prioritize phenotypic stratification and standardized disease classification to improve comparability across studies (Chaiworapongsa et al. 2024).

Several methodological limitations further complicate interpretation of the existing literature. Many clinical studies involve relatively small sample sizes and cross‐sectional designs, limiting statistical power and the ability to establish temporal relationships. Differences in sample collection protocols, laboratory assays, biomarker thresholds, and diagnostic criteria also contribute to variability among findings. Additionally, much of the mechanistic evidence originates from animal models or in vitro experiments that may not fully replicate the complexity of human pregnancy. Although these models provide valuable insights into biological pathways, caution is required when extrapolating findings directly to clinical practice. Biomarker research represents another area where promising findings have not yet translated uniformly into clinical implementation. While markers such as sFlt‐1, PlGF, soluble endoglin, inflammatory cytokines, and endothelial activation markers have demonstrated diagnostic and prognostic potential, challenges remain regarding standardization, cost‐effectiveness, accessibility, and validation across diverse populations. Furthermore, no single biomarker adequately captures the multifaceted nature of pre‐eclampsia, suggesting that future approaches may require integrated biomarker panels reflecting multiple pathogenic pathways. Therapeutic development is similarly constrained by gaps in mechanistic understanding. Although anti‐inflammatory, antiangiogenic, antithrombotic, and endothelial‐targeted interventions have shown promise in experimental settings, robust clinical evidence supporting their routine use remains limited. Many proposed therapies have not undergone adequately powered randomized controlled trials, and concerns regarding maternal‐fetal safety continue to restrict clinical application. Consequently, most emerging therapeutic strategies remain investigational and should be interpreted cautiously until supported by high‐quality clinical data.

## 3 Conclusion

Pre‐eclampsia represents a complex, multisystem disorder of pregnancy that extends beyond the traditional view of a placental disease and is more accurately conceptualized as an integrated immuno‐cardiovascular syndrome. Accumulating evidence indicates that abnormal placentation initiates a cascade of interrelated biological events involving immune dysregulation, cytokine‐mediated inflammation, endothelial dysfunction, thromboinflammation, and maladaptive cardiovascular responses. These processes are not isolated but instead interact dynamically, forming a self‐amplifying network that culminates in the clinical syndrome of hypertension, proteinuria, and multisystem organ dysfunction. Central to this framework is the interaction between inflammatory mediators and vascular biology. Elevated levels of pro‐inflammatory cytokines, antiangiogenic factors, and oxidative stress markers contribute to widespread endothelial injury, impaired vascular reactivity, and activation of coagulation pathways. The resulting immunothrombotic state links immune activation with platelet activation, fibrin deposition, and microvascular injury, thereby providing a mechanistic explanation for both maternal and placental complications. In parallel, cardiovascular maladaptation characterized by increased systemic vascular resistance, impaired cardiac function, and altered hemodynamic responses further exacerbates disease severity and contributes to acute maternal instability.

Pre‐eclampsia is no longer viewed as a uniform disease entity but rather a heterogeneous syndrome encompassing distinct phenotypic subgroups, including early‐onset and late‐onset forms as well as angiogenic and non‐angiogenic variants. This heterogeneity reflects differences in underlying pathophysiological drivers, ranging from severe placental dysfunction to predominant maternal cardiovascular susceptibility. Recognition of these subtypes is essential for improving risk stratification, refining biomarker interpretation, and guiding the development of targeted therapeutic strategies. From a translational perspective, emerging evidence supports the potential utility of integrated biomarker panels, early pregnancy risk prediction models, and phenotype‐specific management strategies. However, the safe and effective implementation of such approaches will require robust clinical validation, standardized methodologies, and careful consideration of maternal–fetal safety. In particular, future therapeutic development must balance modulation of pathological immune and vascular pathways with preservation of essential physiological adaptations required for successful pregnancy. The long‐term implications of pre‐eclampsia extend beyond pregnancy, with affected women demonstrating a significantly increased risk of chronic hypertension, ischemic heart disease, stroke, and heart failure. This reinforces the importance of viewing pre‐eclampsia not only as an acute obstetric complication but also as an early marker of future cardiovascular disease. Accordingly, postpartum surveillance and cardiovascular risk reduction strategies should be integral components of long‐term care.

## Funding

The author has nothing to report.

## Conflicts of Interest

The author declares no conflicts of interest.

## Data Availability

The author has nothing to report.
